# Identification of ferroptosis-related genes as potential diagnostic biomarkers for diabetic nephropathy based on bioinformatics

**DOI:** 10.3389/fmolb.2023.1183530

**Published:** 2023-08-01

**Authors:** Binbin Guo, Minhui Li, Peipei Wu, Yan Chen

**Affiliations:** ^1^ International Special Medical Department, Shengli Oilfield Central Hospital, Dongying, Shandong, China; ^2^ Department of Pediatrics Internal Medicine, Dongying Municipal Children’s Hospital, Dongying, Shandong, China

**Keywords:** diabetic nephropathy, ferroptosis, ferroptosis-related genes, diagnostic biomarkers, *TP53*

## Abstract

**Objective:** This study investigated to probe ferroptosis-related diagnostic biomarkers and underlying molecular mechanisms in Diabetic nephropathy (DN).

**Methods:** GSE30122 and GSE1009 from GEO database were used as training and verification sets, respectively, to screen differentially expressed ferroptosis-related genes (FRGs). These genes were further analyzed using GO, KEGG, and GSEA methods, and screened with PPI, LASSO, and SVM-RFE to identify ferroptosis-related diagnostic biomarkers for DN. A diagnostic model was established using the Glm function and verified with ROC curve. The relationship between these biomarkers and immune cell was analyzed, and qRT-PCR and Western blot were used to detect the expression of these biomarkers in kidney tissues and identify the effect of TP53 on DN development.

**Results:** Fifty one differentially expressed FRGs were enriched in bioprocesses such as *p53* signaling pathway, oxidative stress and chemical stress response, and mTOR signaling pathway. *TP53*, *RB1*, *NF2*, *RRM2*, *PRDX1*, and *CDC25A* were identified as ferroptosis-related diagnostic biomarkers for DN. *TP53* showed the most differential expression. ROC analysis showed that AUC values of *TP53*, *RB1*, *NF2*, *RRM2*, *PRDX1*, and *CDC25A* were 0.751, 0.705, 0.725, 0.882, 0.691, and 0.675, respectively. The AUC value of DN diagnosis model was 0.939 in training set and 1.000 in verification set. qRT-PCR results confirmed significant differences in these six biomarkers between DN and normal kidney tissue (*p* < 0.05), and correlation analysis showed that five biomarkers were significantly correlated with infiltrating immune cells (*p* < 0.05). Furthermore, western blots showed that *TP53* promotes apoptosis through PI3K-AKT signaling in DN.

**Conclusion:** *TP53*, *RB1*, *NF2*, *RRM2*, *PRDX1*, and *CDC25A* have potential as diagnostic biomarkers for DN. The diagnostic model containing the above six biomarkers performs well in the diagnosis of DN. Five of the six biomarkers are strongly associated with several infiltrating immune cells. *TP53* may play an essential role in the development of DN.

## 1 Introduction

Diabetic nephropathy (DN) is a severe complication associated with diabetes and the primary contributor to kidney failure (Sanajou et al., 2018; Sagoo and Gnudi, 2020). It is characterized by progressive renal impairment, deterioration of glomerular filtration rate (GFR), elevated serum creatinine level (SCR), hypertension, and high mortality (Giralt-López et al., 2020; Zhou et al., 2022). However, the specificity and reliability of these two indicators are limited (Ali et al., 2022). In recent years, researchers have explored various biomarkers and molecular pathways associated with the development and progression of DN (Pentyala et al., 2015; Liang et al., 2022). One area of interest in DN is the investigation of ferroptosis.

Ferroptosis is a form of cell death, has gained significant attention in recent years due to its involvement in various pathological conditions, including DN (Huang et al., 2022). The initiation of ferroptosis involves the accumulation of lipid peroxides and a decrease in the activity of glutathione peroxidase 4 (GPX4). Overexpression of ferritin 1 and transferrin receptor 1(TfR1) results in excessive buildup of ferrous ions, which results in generation of excessive reactive oxygen species (ROS) (Kajarabille and Latunde-Dada, 2019). Another critical aspect of ferroptosis in DN is the disruption of the cystine/glutamate amino acid transport system. Researches have showed that the dysfunction of this transport system impairs the antioxidant role of GPX4, rendering the cell membrane vulnerable to ROS attack and subsequent lipid peroxidation (Su et al., 2019; Wang et al., 2020b). Characteristic ferroptosis changes in animal models of DN, such as ROS accumulation, decreased antioxidant capacity, and lipid peroxidation product accumulation (Dixon et al., 2012; Dixon and Stockwell, 2014; Keller et al., 2016; Wang et al., 2020a). Moreover, Li et al. (2021) validated that inhibition of ferroptosis could delay DN progression in diabetic mice. In DN patients, ferroptosis-related molecules, such as long-chain acyl-CoA synthetase 4 (ACSL4) and GPX4, exhibit abnormal changes (Wu et al., 2021). Wang et al. (2020c) also found abnormal changes in ACSL4 through DN mouse model, and found that inhibition of ACSL4 could block ferroptosis of renal tubular cells and alleviate DN symptoms. Therefore, targeting ferroptosis-related pathways and molecules holds promise for the development of novel therapeutic strategies to mitigate DN progression and preserve renal function. Bioinformatics analysis has emerged as a powerful tool in disease research, aiding in the identification of potential biomarkers and therapeutic targets. In recent years, several studies have utilized bioinformatics approaches to gain insights into various diseases (Qiu et al., 2021; Wang et al., 2021; Xu and Chen, 2021). In the context of DN, Geng et al. (2019) used bioinformatics analysis to identify eight core genes in DN, among which *Itgb2* contributed to DN by promoting the transcription of *EST1*. Wu et al. (2022) revealed the ferroptosis-related gene (FRG) *HMOX1* as a potential diagnostic biomarker of atherosclerosis using bioinformatics analysis. However, few people have explored the ferroptosis-related diagnostic biomarkers of DN through bioinformatics analysis.

This study has focused on the identification of diagnostic biomarkers associated with ferroptosis and the elucidation of molecular mechanisms that drive the progression of DN. These investigations have enhanced our comprehension of the role played by ferroptosis in DN development, thus establishing a novel theoretical foundation for the treatment of DN.

## 2 Objects and methods

### 2.1 Data download and pre-processing

The Gene Expression Omnibus (GEO) database (https://www.ncbi.nlm.nih.gov/geo) served as the primary resource for obtaining microarray datasets pertaining to diabetic nephropathy. This comprehensive database offers a vast collection of freely accessible microarray, RNA-seq, and other pertinent data, making it an invaluable tool in the domains of genetics and bioinformatics. By employing the advanced search functionality, we identified microarray profiles that included the keywords “diabetic nephropathy” and “*Homo sapiens*” within their titles or abstracts. Among them, the GSE30122 dataset consisted of 50 normal kidney tissue samples (control group) and 19 DN kidney tissue samples (DN group), while the GSE1009 dataset consisted of 3 normal kidney tissue samples (control group) and 3 DN kidney tissue samples (DN group). Subsequently, we downloaded the GSE30122 and GSE1009 datasets from the GEO database utilizing the “GEOquery” package. The GSE30122 dataset serves as the training set, while the GSE1009 dataset functions as the validation set. The FRGs were obtained from FerrDb (http://www.zhounan.org/ferrdb) database.

### 2.2 Differential expression analysis

We used the “limma” package to perform differential expression analysis on the batch-corrected CHD datasets. The threshold for identifying differentially expressed genes (DEGs) was set at |log2FC| > 1 and *p* < 0.05.

### 2.3 GO and KEGG pathway enrichment analysis

GO and KEGG pathway enrichment analyses were performed on the differentially expressed FRGs using the clusterProfiler package. Enrichment analyses with *p* < 0.05 were considered statistically significant.

### 2.4 PPI analysis

To identify the key significant FRGs with differential expression, we constructed a PPI network based on the STRING database (http://string-db.org). We used Cytoscape (v 3.9.1) to visualize the resulting network, and identified hub genes through plugins in Cytoscape.

### 2.5 Identification and evaluation of diagnostic biomarkers for DN

To further refine the list of hub genes, we used LASSO regression model from the glmnet package (Bălăşescu et al., 2015). We determined the optimal parameter (λ) using 10-fold cross-validation and plotted the partial likelihood deviation curves relative to log(λ). We also used the SVM-RFE method from the e1071 package to narrow down the hub genes (Pandey et al., 2019). The intersection of the results from both methods yielded ferroptosis-related diagnostic biomarkers for DN. We evaluated the diagnostic performance of these biomarkers using ROC curves.

### 2.6 Expression and validation of diagnostic biomarkers for DN

The ggpubr package was utilized to create visualizations of the expression levels of six identified biomarkers in the training set. We collected 10 DN tissue samples and 10 normal kidney tissue samples from diagnostic kidney biopsies at our hospital. The study was approved by our hospital’s ethics committee, and all participants provided informed consent. The expressions of *TP53*, *RB1*, *NF2*, *RRM2*, *PRDX1*, and *CDC25A* were quantified using qRT-PCR according to the manufacturer’s instructions. We used the 2^−ΔΔCt^ method, with *GAPDH* as an internal control.

### 2.7 Analysis of immune cell infiltration

We used CIBERSORT package to obtain an immune cell infiltration matrix for 22 types of immune cells in control and DN samples. The violin diagram was used to visualize the differences between the two groups.

### 2.8 Cell culture

Dr. Moin Saleem provided us with an immortalized human podocyte cell line. These cells were cultured in RPMI-1640 (Thermo Fisher, Waltham, MA, United States) supplemented with 10% fetal bovine serum (T Gibco, Rockville, MD, United States) and 100 U/mL penicillin mixture (Thermo Fisher, Waltham, MA, United States) at 37°C and 5% CO_2_.

### 2.9 Cell transfection

Plasmid directly against *TP53*-silenced small interfering RNA (siRNA) and corresponding negative control were purchased from Guangzhou Ruibo Biotechnology (Guangzhou, Guangdong, China). Lipofectamine™ 2000 was used to transfect the corresponding plasmid or siRNA into human podocyte cell line.

### 2.10 Western blotting

Protein extraction was performed using RIPA buffer (Shanghai Life Mode Engineering, Shanghai, China) supplemented with PMSF (Shanghai Life Mode Engineering, Shanghai, China). The primary antibody (Table 1) was incubated with a PVDF membrane (0.22 μm, Millipore ISEQ00010, United States) at 4°C overnight, followed by incubation with a secondary antibody (1:2000, Abcam, Waltham, MA, United States) conjugated to HRP. Protein bands were visualized using Prime Western blotting detection reagent (Cytiva, United Kingdom), and the chemiluminescence was detected using a ChemiDoc MP imaging system (Tanon 4800, Shanghai, China). The gray value of the bands was analyzed using ImageJ software.

**TABLE 1:** All antibodies in this study.

Gene	Brand	Provenance
PI3K	Abcam	Rabbit
AKT3	Abcam	Rabbit
p-AKT	Abcam	Rabbit
mTOR	Abcam	Rabbit
p-mTOR	Abcam	Rabbit
PARP	Abcam	Rabbit
c-PARP	Abcam	Rabbit
Casepase-3	Abcam	Rabbit
C-Casepase-3	Abcam	Rabbit
GADPH	Sigma	Mouse

### 2.11 Statistical analysis

Data were statistically analyzed by R 4.2.1 or GraphPad Prism 9.0. Unpaired t-test was used for comparison between the two groups. ROC analysis was applied to evaluate the diagnostic ability of a single biomarker or model. Correlation analysis was performed by spearman correlation. *p* < 0.05 was considered statistically significant.

## 3 Results

### 3.1 Data normalization and differential expression genes (DEGs) analysis

**FIGURE 1:**
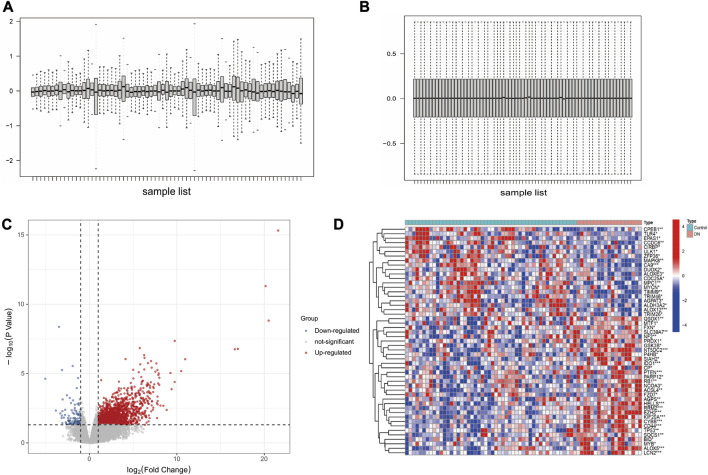
Data preprocessing and DEGs screening. **(A)** DN datasets before standardization. **(B)** DN datasets after standardization. **(C)** Volcano plot analysis of DEGs. **(D)** The heatmap of 51 differentially expressed FRGs.

The GSE30122 dataset was downloaded from the GEO database, and its normalization was shown in Figures 1A, B. Preprocessing of the dataset resulted in the identification of 392 DEGs, with 188 genes upregulated and 204 genes downregulated (Figure 1C). FRGs were obtained from the GeneCards database. According to GeneCards database, 51 FRGs were identified as DEGs (Figure 1D).

### 3.2 GO and KEGG analysis of differentially expressed FRGs

**FIGURE 2:**
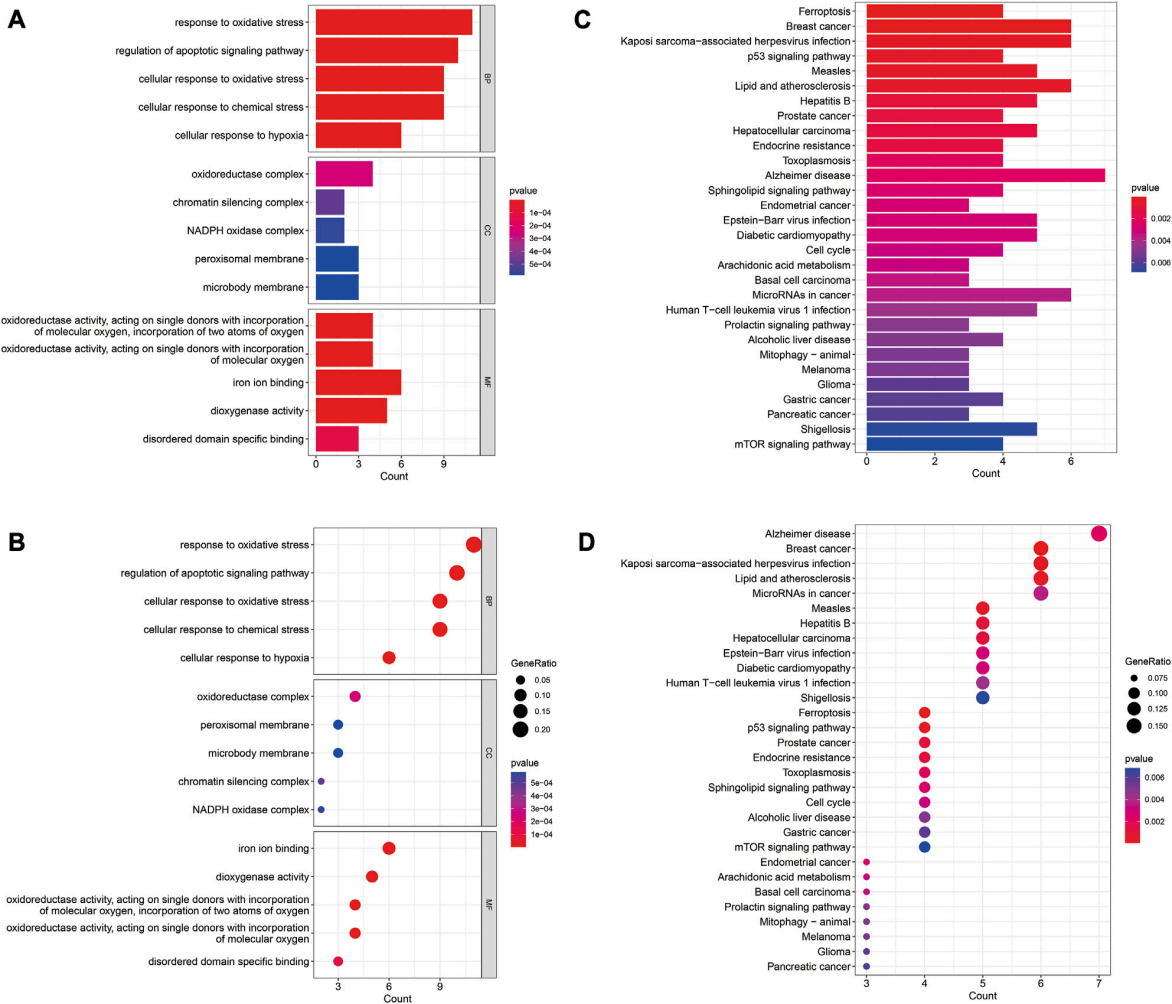
GO and KEGG enrichment analysis of differentially expressed FRGs. **(A)** Barplot of GO enrichment analysis. **(B)** Barplot of KEGG pathway analysis. **(C)** Dotplot of GO enrichment analysis; **(D)** Dotplot of KEGG pathway analysis.

The GO enrichment results revealed that these genes were mainly involved in regulation of apoptotic signaling pathway, cellular response to oxidative stress, and chemical stress (Figures 2A, B). KEGG analysis showed these genes were mainly associations with lipids and atherosclerosis, *p53* signaling pathway, and mTOR signaling pathway (Figures 2C, D).

### 3.3 GSEA analysis of differentially expressed FRGs

**FIGURE 3:**
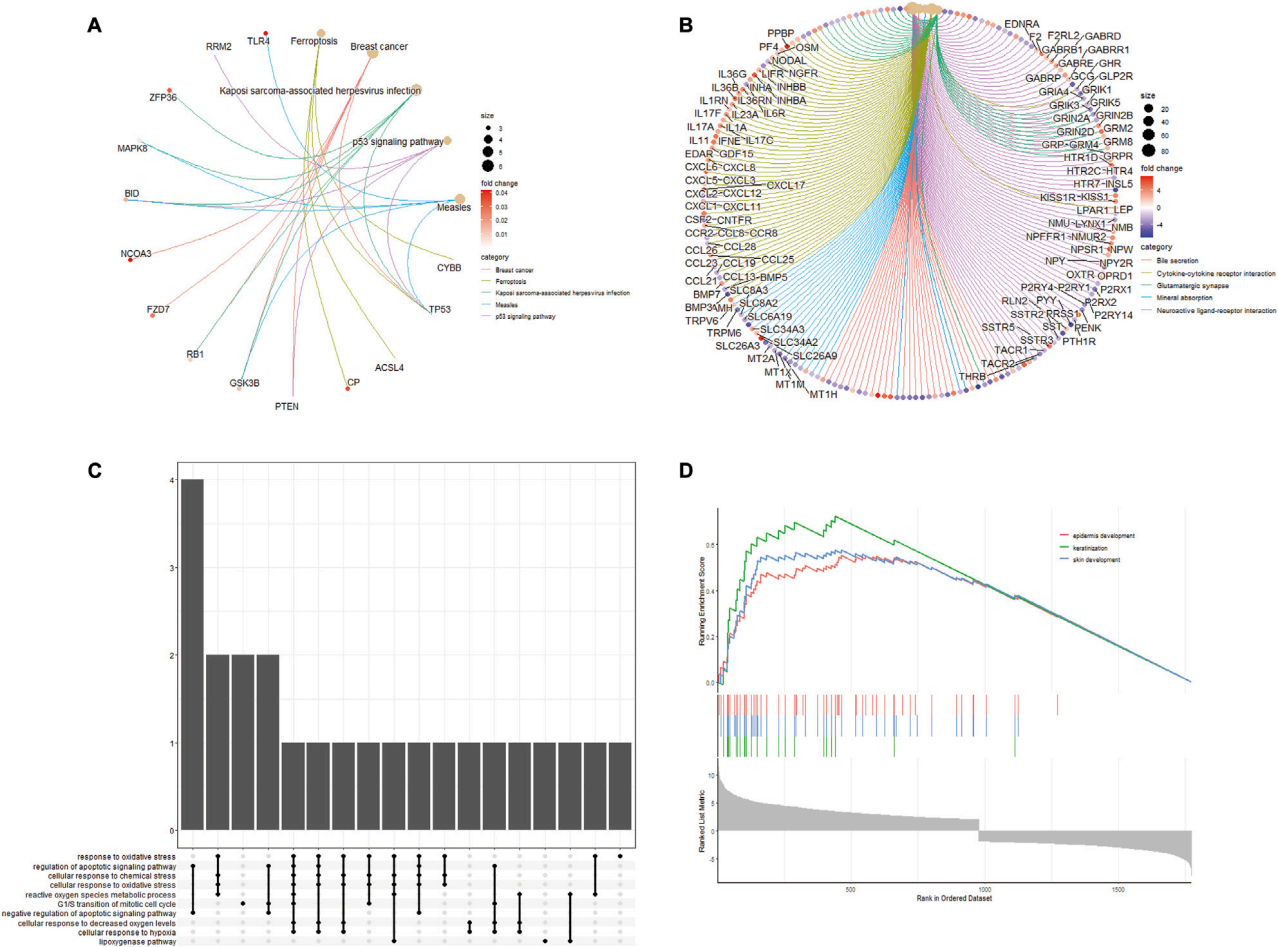
GSEA enrichment analysis of differentially expressed FRGs. **(A)** Enrichment plot of KEGG entries of differentially expressed FRGs. **(B)** Enrichment plot of GO entries. **(C)** UpSetR plot of the GO annotations in KEGG. **(D)** Top3 enrichment of GSEA analysis for differentially expressed FRGs.

As shown in Figures 3A, B We found that multiple biological pathways were significantly altered in DN kidney tissues compared with the control kidneys by GSEA. Using R package “UpSetR,” we investigated modules related to KEGG pathways (Figure 3C). “Epidermis development,” “keratinization,” and “skin development” were Top3 enriched pathway (Figure 3D).

### 3.4 PPI analysis of differentially expressed FRGs

**FIGURE 4:**
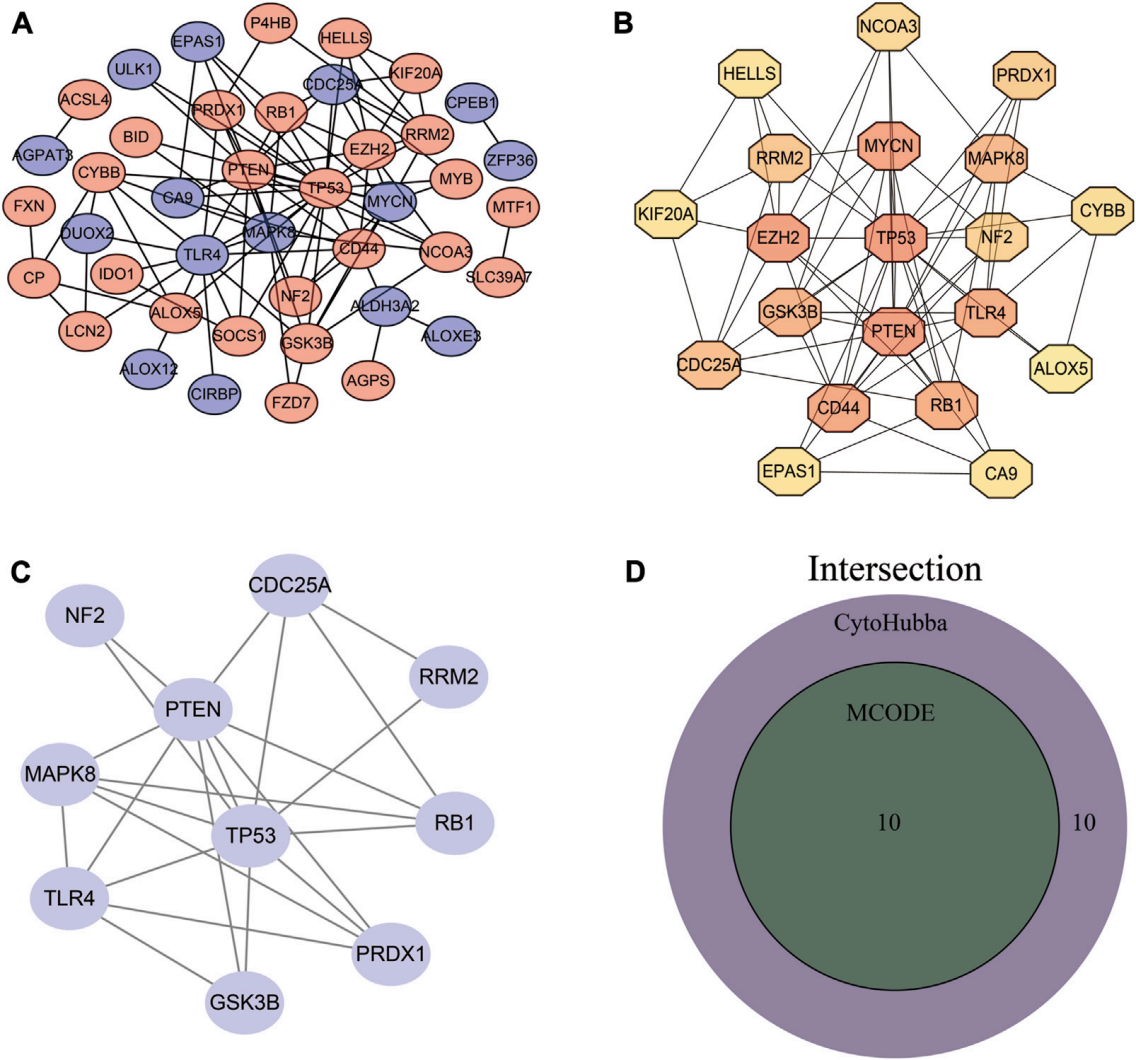
PPI network establishment and hub gene identification. **(A)** PPI network. **(B)** Hub genes obtained by MCODE plug-in analysis. **(C)** Top 20 genes obtained by MCC algorithm. **(D)** Venn diagram of MCODE and CytoHubba results.

To better clarify interactions between differentially expressed, we performed PPI network analysis using the STRING database (Figure 4A). We then applied two plugins from Cytoscape, MCODE, and CytoHubba, to screen the genes (Figures 4B, C). After the intersection of the two results, 10 differentially expressed FRGs were identified as hub genes (Figure 4D).

### 3.5 Identification and evaluation of DN diagnostic biomarkers

**FIGURE 5:**
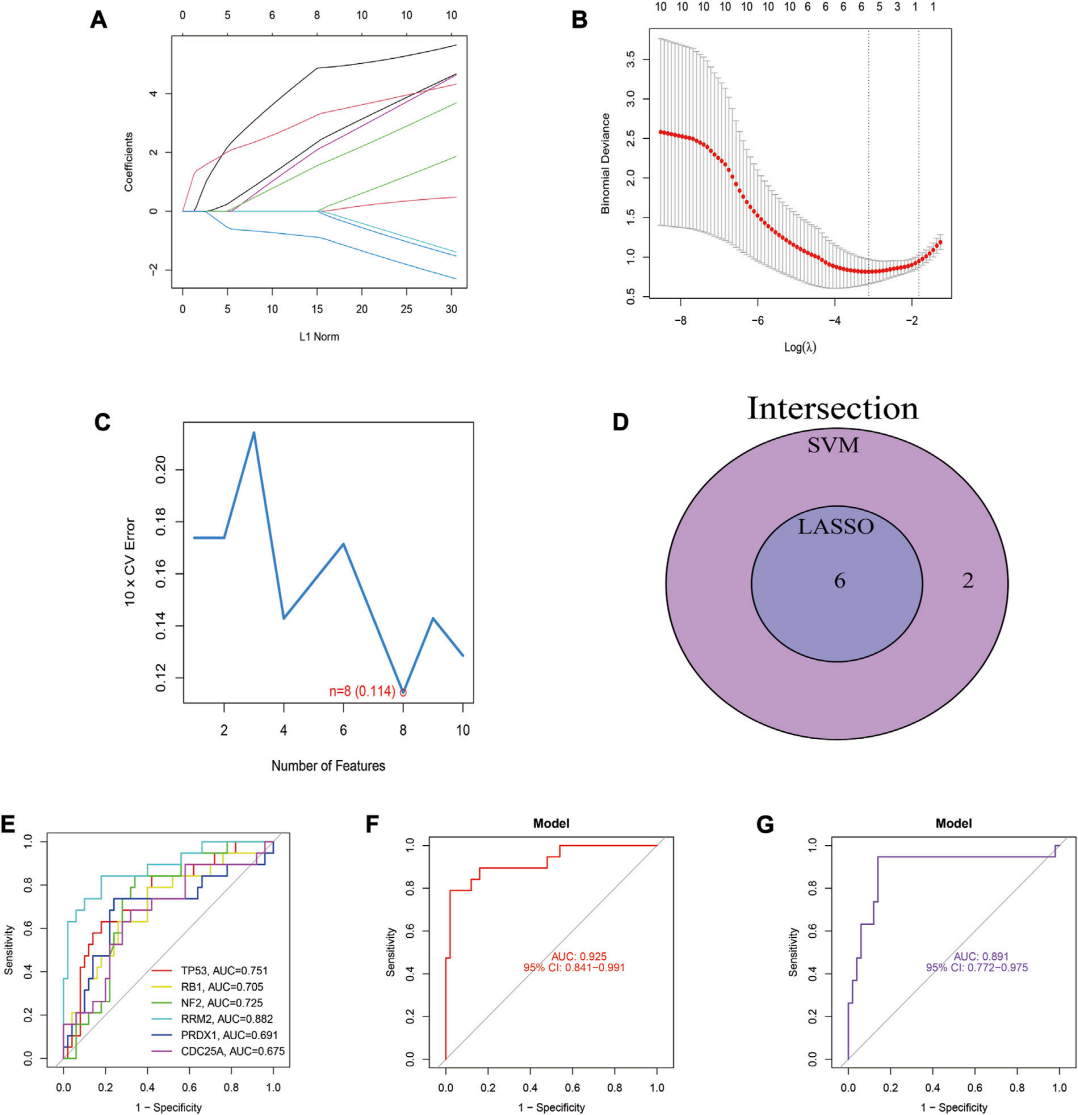
Identification and evaluation of biomarkers for DN. **(A)** LASSO regression of 10 hub genes. **(B)** Cross validation of parameter selection in LASSO regression. **(C)** The important feature selection graph obtained by SVM-RFE algorithm. **(D)** Venn diagram of LASSO regression and SVM-RFE results. **(E)** ROC curves of 6 diagnostic biomarkers for DN. **(F)** The ROC curve for this diagnostic model in the training set. **(G)** The ROC curve for this model in the validation set.

LASSO regression and SVM-RFE were utilized to screen hub genes to identify diagnostic biomarkers for DN. Six important variables were obtained by LASSO regression (Figures 5A, B). Eight features were obtained using SVM-RFE algorithm (Figure 5C). After taking the intersection of the results obtained by these two methods, six overlapping hub genes were obtained (Figure 5D). The six hub genes were *TP53*, *RB1*, *NF2*, *RRM2*, *PRDX1*, and *CDC25A*. Figure 5E shows the evaluation results of the six hub genes, and the AUC values of *TP53*, *RB1*, *NF2*, *RRM2*, *PRDX1*, and *CDC25A* were 0.751, 0.705, 0.725, 0.882, 0.691, and 0.675, respectively. Finally, we identified these six genes as the ferroptosis-related diagnostic biomarkers of DN. *TP53*, *RB1*, *NF2*, *RRM2*, *PRDX1*, and *CDC25A* were established as a diagnostic model for DN. The diagnostic model was evaluated using ROC curves, with an AUC value of 0.939 (95% CI: 0.863–0.993) in the training set, and an AUC of 1.000 (95% CI: 1.000–1.000) in the validation set (Figures 5F, G).

### 3.6 Association of DN diagnostic biomarkers with infiltrating immune cells

**FIGURE 6:**
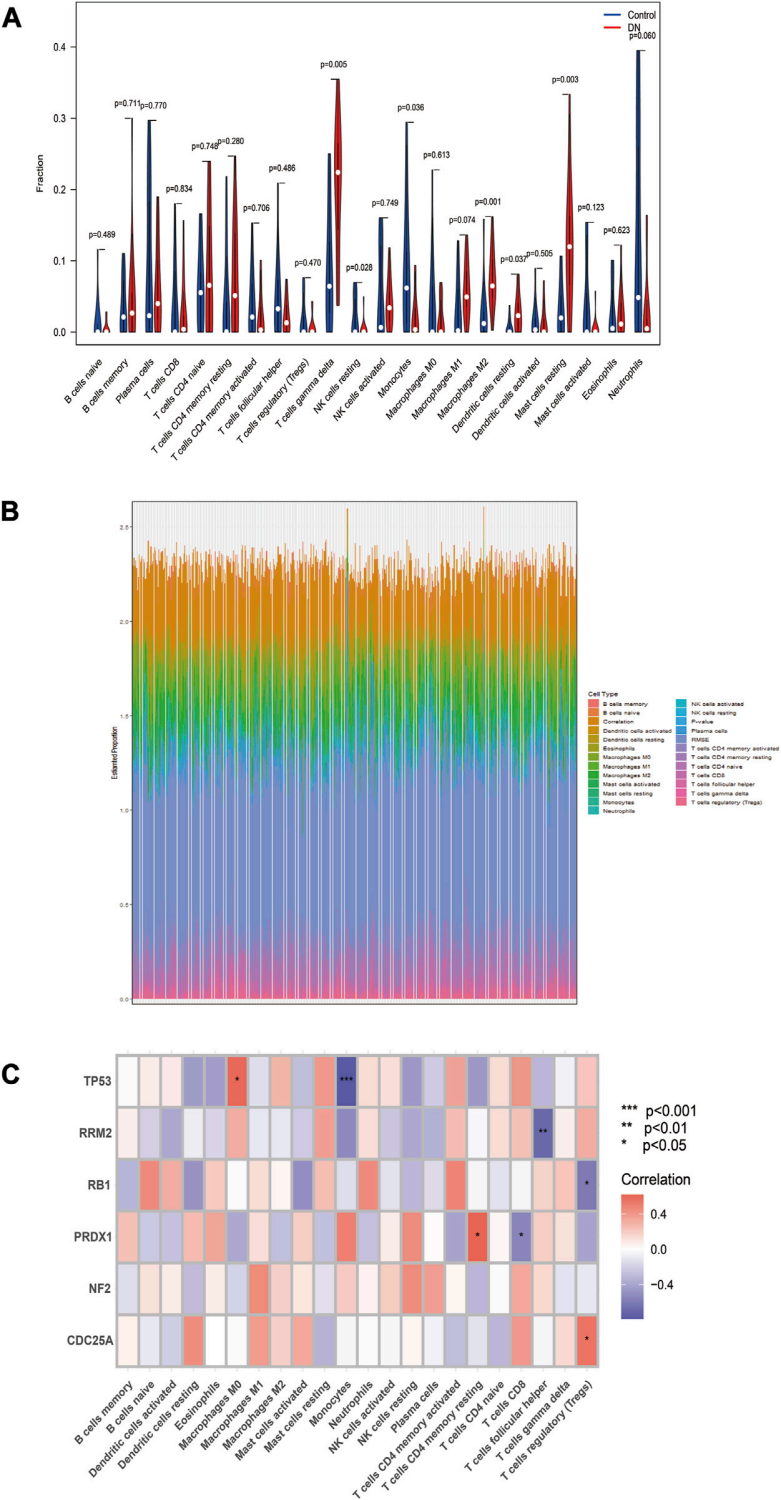
Evaluation of immune cell infiltration. **(A)** Differences in immune cell infiltration between the two groups. **(B)** Waterfall plot for the percentage of immune cell. **(C)** Relationship between six biomarkers and immune cell infiltration in the same DN samples. *p < 0.05, **p < 0.01, ***p < 0.001, compared with Control group.

The study examined infiltrating immune cells in control and DN samples and found significant differences in immune cells (Figures 6A, B). Next, we investigated the relationship between immune cell infiltration and *TP53*, *RB1*, *NF2*, *RRM2*, *PRDX1*, and *CDC25A* in DN samples (Figure 6C).

### 3.7 Expression and validation of these six biomarkers

**FIGURE 7:**
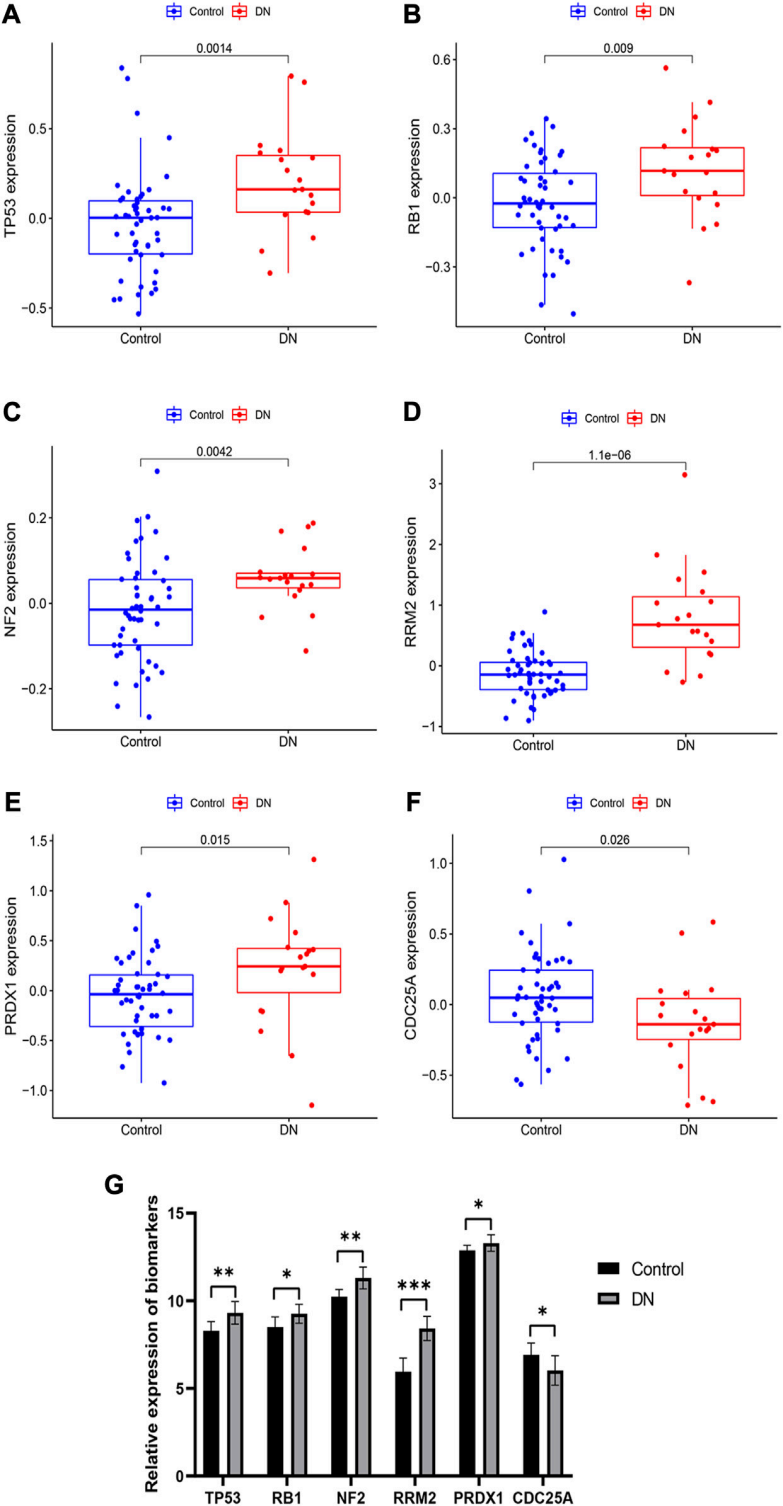
Expression and validation of these six diagnostic markers. **(A–F)** expression of these 6 biomarkers in the training set. **(G)** Expression of these 6 biomarkers obtained by qRT-PCR. *p < 0.05, **p < 0.01, ***p < 0.001, compared with Control group.

Box plots were used to visualize the expression levels of these six biomarkers in the training set. Figures 7A–F illustrates that *TP53*, *RB1*, *NF2*, *RRM2*, and *PRDX1* expression levels were significantly increased in the DN group compared to the control group. Conversely, *CDC25A* expression level was significantly decreased in the DN group. To validate the results, we detected their expression levels in human normal kidney tissues and DN tissues by qRT-PCR. The results shown in Figure 7G were consistent with those in the training set.

### 3.8 TP53 promoted cell apoptosis in DN through PI3K-AKT signaling pathway

**FIGURE 8:**
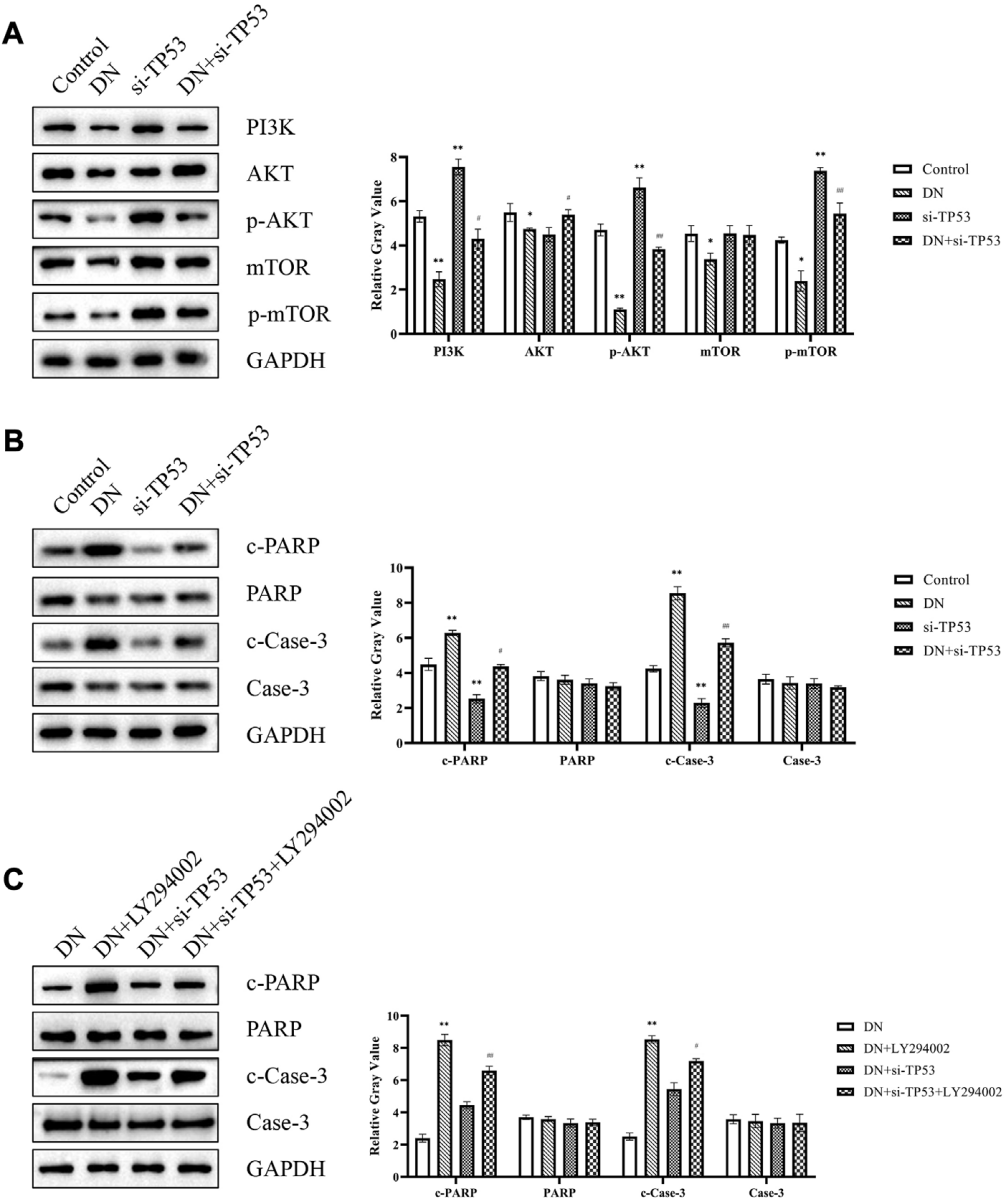
TP53 promoted DN through PI3K-AKT signaling pathway. **(A)** Western blot analysis of signal proteins in the PI3K-AKT signaling pathway. **(B,C)** Western blot analysis of cell apoptosis-related genes. *p < 0.05, **p < 0.01, compared with the Control group; ^#^ *p* < 0.05, ^##^ *p* < 0.01, compared with the *si-TP53* group; ^++^ *p* < 0.01, compared with the DN group; ^^^ *p* < 0.05, ^^^^ *p* < 0.01, compared with the DN + *si-TP53* group.

*TP53* was the greatest fold change difference gene in diagnostic markers of DN. Thus, we next explored the effect of *TP53* in DN. Western blot analysis to determine the expression of signal proteins in the *PI3K*-*AKT* signaling pathway, including *PI3K*, *AKT*, *p-AKT*, *mTOR*, and *p-mTOR*. The results indicated that *PI3K*-*AKT* signaling was activated in the *si-TP53* group and DN + *si-TP53* group compared to the control and DN groups, respectively (Figure 8A). Additionally, si-TP53 significantly increased the expression of *c-PARP* and *c-Case-3* compared to the DN group (Figure 8B). We further inhibited the *PI3K*-*AKT* signaling pathway using LY294002 and observed significant inhibition of cell apoptosis in contrast to the DN group. However, *si-TP53* reversed the process of cell apoptosis in the DN-*si-TP53* + LY294002 group (Figure 8C). The intact original pictures of Western blot were shown in Supplementary Figure S1.

## 4 Discussion

Multiple studies have found that ferroptosis plays a crucial role in various diseases (Li et al., 2020; Jiang et al., 2021). Recently, bioinformatics analysis has been playing a crucial role in disease studies and facilitates the understanding of cellular and molecular mechanisms behind the progression of disease (Deng et al., 2021; Kazeminasab et al., 2021). The present study performed bioinformatics to analyze the diabetes data sets from GEO database to confirm the key diabetes-causing genes and determine their significance in diabetes prognosis.

We conducted a DEGs analysis of GSE30122, which included 50 control samples and 19 DN samples. Among the 191 DEGs we identified, 51 were FRGs. The GO enrichment analysis revealed that these 51 FRGs were mainly enriched in cell apoptosis, cell response to oxidative and chemical stress, lipids and atherosclerosis. Persistent metabolic abnormalities caused by diabetes will lead to cell signal transduction imbalance and cell turnover disorder through the pro-apoptotic pathway, thereby accelerating the progression of DN (Ryan et al., 2009; Bălăşescu et al., 2015; Pandey et al., 2019). Prolonged state of Hyperglycemia is a condition that lead to oxidative stress, produce too much ROS, severely damage the body function of antioxidant, lead to imbalance between oxidation and antioxidant systems, and ultimately results in kidney impairment (Negi et al., 2011; Ganesh Yerra et al., 2013; Areti et al., 2016). As is well known, p53 signaling pathway is apoptotic signaling pathway, *p53* positively regulates *Bax* (pro-apoptotic protein) and negatively regulates the transcription of Bcl-2 (anti-apoptotic protein) (Zhu et al., 2020). Ren et al. (2017) found that *p53* pathway regulated cell apoptosis and played a vital role in Type 2 Diabetes Mellitus (T2DM). In the kidney of DN patients, the *mTOR* signaling pathway was inhibited and subsequently suppressed the autophagy, caused mesangial expansion and proliferation, eventually led to glomerular hypertrophy and kidney damage (Lu et al., 2019).

PPI network was conducted on 51 FRGs and screened 10 hub genes. Next, we identified six diagnostic biomarkers for DN by lasso regression and support vector machine analysis, including *P53*, *RB1*, *NF2*, *RRM2*, *PRDX1*, and *CDC25A*. In order to validate the accuracy of the diagnostic models, we applied GSE1009 and used ROC curve analysis. The AUC value of the diagnostic model in the training set was 0.939 in the GSE30122, and the AUC of 1.000 in the GSE1009, indicating a promising predictive performance of the DN diagnostic model.

*TP53* is a tumor suppressor gene and participates in a variety of biological processes. Prior research has indicated that activating the *TP53* pathway can heighten vulnerability to apoptosis, particularly in the context of Type 1 Diabetes Mellitus (T1DM) (Bernard et al., 2020; Lacroix et al., 2020; Punja et al., 2021). Our qPCR results showed a significant increase in TP53 expression levels in DN tissues. *RB1* has also been implicated in inflammatory diseases (Dyson, 2016; Muñoz-Fontela et al., 2016), and our study showed that its expression was upregulated in DN tissues. Furthermore, our study found that peroxiredoxin 1 (PRDX1) was significantly overexpressed in DN samples, which is consistent with previous studies reporting higher plasma PRDX1 levels in T2DM patients (Tang et al., 2015). We also identified *NF2* and *RRM2* as highly expressed genes in DN samples, while *CDC25A* was found to be lowly expressed. Our results showed that both *RRM2* and *CDC25A* were associated with T cells, although no previous studies have implicated *NF2*, *RRM2*, and *CDC25A* in the process of DN.

Among all DEGs, *TP53* is the gene with the greatest expression differences. Thus, we next explored its potential regulatory pathway in DN. The *PI3K*/*AKT* signaling pathway is one of the critical pathways involved in regulating cell growth and apoptosis. Phosphatidylinositol 3-kinase (PI3K) is the important cytokine of this pathway, which has phosphatidylinositol kinase. With the dual activity of serine/threonine protein kinase, *PI3K* will be converted into the second messenger *PIP3* after activation, and *PIP3* will then activate *AKT* to *p-AKT*. *p-AKT* can block the combination of Bad and Bcl-2, and inhibits the occurrence and development of apoptosis (LoPiccolo et al., 2008; Wang et al., 2015). Western blot analysis of the DN + *si-TP53* group showed that the *PI3K*-*AKT* signaling pathway was activated and that hallmark apoptosis markers, *PARP* and *caspase-3*, were also activated compared to the DN group. LY294002 is a commonly used *PI3K*/*AKT* pathway inhibitor (Nie et al., 2019). Our results demonstrated that treatment with LY294002 could significantly rescued *si-TP53*-mediated cell apoptosis.

In summary, our analysis of DN data from the GEO database using bioinformatic tools led to the identification of potential molecular targets related to ferroptosis in DN. Furthermore, we developed a 6-gene model related to ferroptosis, which demonstrated excellent predictive performance for DN. Finally, qPCR and western blot analyses confirmed that *TP53* promotes cell apoptosis through the *PI3K*-*Akt* pathway, ultimately leading to DN.

## Data Availability

The original contributions presented in the study are included in the article/Supplementary Materials, further inquiries can be directed to the corresponding authors.

## Abbreviations

DN, Diabetic nephropathy; ACSL4, acyl-CoA synthetase 4; PRDX1, peroxiredoxin 1; T1DM, Type 1 Diabetes Mellitus; DEGs, differentially expressed genes; FRG, ferroptosis-related gene; FRGs, ferroptosis-related genes; GFR, glomerular filtration rate; GPX4, glutathione peroxidase 4; PI3K, phosphatidylinositol 3-kinase; ROS, reactive oxygen species; SCR, serum creatinine level; si RNA, small interfering RNA; T2DM, Type 2 Diabetes Mellitus; TfR1, transferrin receptor 1.
