# Supplementary material for: Identification of ferroptosis-related genes as potential diagnostic biomarkers for diabetic nephropathy based on bioinformatics
Source: Front Mol Biosci. 2023 Aug 1;10:1183530. doi: 10.3389/fmolb.2023.1183530 (PMC10428009; doi:10.3389/fmolb.2023.1183530)

**Identification of Ferroptosis-related genes as potential diagnostic biomarkers for Diabetic nephropathy Based on Bioinformatics**

**Binbin Guo ^1,†^, Minhui Li ^2,†^, Peipei Wu ^1,*^, Yan Chen ^1,*^**

*^1^* *International Special Medical Department, Shengli Oilfield Central Hospital, No. 31 Jinan Road, Dongying City 257000, Shandong Province, China;*

*^2^ Department of Pediatrics Internal Medicine, Dongying Municipal Children's Hospital,317 Nanyi Road, Dongying City257091, Shandong Province, China*

*^†^ These authors contributed equally to this work.*

*** Correspondence:**Yan Chen, yanchenslyt@163.com; Peipei Wu, peipeiwu2023@163.com.

**Supplementary file**

**Figure S1. The intact original pictures of Western blot.** (A) The intact original pictures with full markers of Figure 8A, (B)The intact original pictures with full markers of Figure 8B, (C) The intact original pictures with full markers of Figure 8C


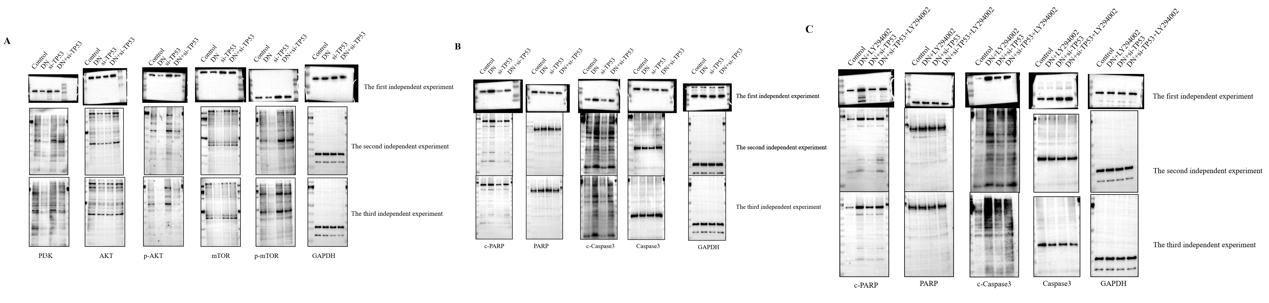

Supplement: Supplementary file 1 [file DataSheet1.DOCX]
